# A systematic review of gerontechnologies to support aging in place among community-dwelling older adults and their family caregivers

**DOI:** 10.3389/fpsyg.2023.1237694

**Published:** 2024-01-24

**Authors:** Alexander Moreno, Maria-Cristina Scola, Hua Sun, Henrick Durce, Célia Couve, Kelly Acevedo, Gloria M. Gutman

**Affiliations:** ^1^Department of Psychology, Université de Montréal, Montréal, QC, Canada; ^2^Notre-Dame Hospital, Centre Intégré Universitaire de Santé et de Services Sociaux du Centre-Sud-de-l'Île-de-Montréal (CCSMTL), Montréal, QC, Canada; ^3^Centre de Recherche de l’Institut Universitaire de Gériatrie de Montréal, CIUSSS du Centre-Sud-de-l’Île-de-Montréal, Montréal, QC, Canada; ^4^Department of Psychoeducation, Université de Montréal, Montréal, QC, Canada; ^5^Gerontology Research Centre, Simon Fraser University, Vancouver, BC, Canada

**Keywords:** Gerontechnology, AgeTech, Community-Dwelling Older Adults (CDOA), Family Caregivers (FC), Aging in place, Home support, Older adult, Aging

## Abstract

**Objective:**

Paucity of information concerning the efficacy of gerontechnologies to support aging in place among community-dwelling older adults prevents potential users, healthcare professionals, and policymakers from making informed decisions on their use. The goal of this study was to identify gerontechnologies tested for home support in dyads of community-dwelling older adults with unimpaired cognition and their family caregivers, including their benefits and challenges. We also provide the level of evidence of the studies and recommendations to address the specific challenges preventing their use, dissemination, and implementation.

**Methods:**

We conducted a systematic review of the literature published between 2016 and 2021 on gerontechnologies tested for home support in dyads. Two independent reviewers screened the abstracts according to the inclusion/exclusion criteria. A third reviewer resolved eligibility discrepancies. Data extraction was conducted by two independent reviewers.

**Results:**

Of 1,441 articles screened, only 13 studies met the inclusion criteria with studies of moderate quality. Mostly, these gerontechnologies were used to monitor the older adult or the environment, to increase communication with family caregivers, to assist in daily living activities, and to provide health information. Benefits included facilitating communication, increasing safety, and reducing stress. Common challenges included difficulties using the technologies, technical problems, privacy issues, increased stress and dissatisfaction, and a mismatch between values and needs.

**Conclusion:**

Only a few gerontechnologies have proven efficacy in supporting community-dwelling older adults and their family caregivers. The inclusion of values and preferences, co-creation with end users, designing easy-to-use technologies, and assuring training are strongly recommended to increase acceptability and dissemination.

**Systematic review registration:**

https://www.crd.york.ac.uk/prospero/display_record.php?RecordID=310803, identifier CRD42022310803.

## Introduction

In the past decades, the world population has shown a steady increase in the proportion of individuals aged 65 years and older. Approximately 10% of the world’s population is comprised of older adults (United Nations, 2021). This proportion is approximately double in Canada (18%) (StatCan, 2022) and other developed countries. This population trend poses worldwide challenges in the management of the health and well-being of older adults and their family caregivers.

Older adults prefer to live independently in their homes rather than in alternative living arrangements such as assisted living or long-term care (Kim, 2021). Independent living goes beyond a mere preference since it is linked to increased engagement with care service providers, self-determination, participation in problem-solving daily challenges, and improvement of mental health (Hurstfield et al., 2007). Considering these important benefits, governments around the world are shifting their policies to fund home and community-based services for older adults.

Older adults living with physical or cognitive difficulties may choose to move to a long-term care facility when they realize that there may be important risks for their health. Most older adults move to long-term care because they can no longer manage or be managed at home, and it is often someone else who makes the decision. The risk of social isolation, malnutrition, falls or other accidental injuries, as well as physical and cognitive deconditioning are important factors to consider (Moreland et al., 2012; Crichton et al., 2019; Fakoya et al., 2020; Di Lorito et al., 2021). For family caregivers, overload, burden, and compassion fatigue associated with high levels of chronic stress should be prevented or treated (Alves et al., 2019; Liao et al., 2022).

Different stakeholders including researchers, healthcare professionals, and the industry have been striving to develop technologies that can support older adults at home to promote their independence and autonomy. Gerontechnology is a transdisciplinary field using technologies (systems and equipment) to promote healthy aging and to solve problems related to chores, leisure, communication, and safety (Halicka, 2019). Gerontechnologies are used to prevent, delay, or compensate for physical, cognitive, and sensorial decline due to aging. For instance, gerontechnologies are used to optimize communication with family caregivers, monitor older adults and the environment to increase safety, and to assist in daily living activities (Colnar et al., 2020). At the same time, older adults can face barriers using technologies, particularly when they experience cognitive decline (Ikeda et al., 2021). For this reason, early intervention, familiarization, and progressive adaptation of these technologies can increase their impact. Family caregivers play an important role in the development, selection, and adoption of technologies to improve care in older adults (Leslie et al., 2021). A current paucity of information about the efficacy of these technologies prevents older adults, family caregivers, healthcare professionals, and policymakers from making informed decisions about their use.

## Objective and research questions

The objective of this study was to evaluate and synthetize information via a systematic review of literature published between 2016 and 2021 concerning gerontechnologies used for home support among Community-Dwelling Older Adults (CDOA) without cognitive impairment and their Family Caregivers. The systematic review was designed to answer four main questions:

1. What gerontechnologies have been tested for home support by both CDOA and their family caregivers?
2. What are the benefits, challenges, and opportunities provided by these gerontechnologies for CDOA and their family caregivers?
3. What is the evidence level of the studies conducted with dyads comprised of CDOA and their family caregivers?
4. What recommendations, if any, address the specific challenges preventing the use and dissemination of these gerontechnologies?

## Methods

### Search strategy and information sources

We followed the Preferred Reporting Items for Systematic Reviews and Meta-Analyses (PRISMA). A systematic review of literature published between 2016 and 2021 was conducted by the principal investigator (AM) in collaboration with two librarians with a background in Psychology and Geriatrics. The two librarians participated in different iterations and validations of the search strategy. Databases searched included: CINAHL, Medline, PsycINFO, Web of Science, and AgeLine. The search terms included « home support », « older adults », « family caregivers », and « technology ». Table 1 presents the search strategy, as well as truncation symbols (denoted by *) and Boolean operators (AND, OR). The systematic review was registered in PROSPERO (registration number: CRD42022310803).

**Table 1:** Search terms and results from each database.

**Database**	**Search strategy**	**References**
PsycINFO	(((home adj2 care) or own home or (living adj2 independent*) or (aging adj2 independent*) or (base adj2 home) or community dwelling or living alone or aging in place).mp or Home Care/or Living alone/or Home Environment/or Aging in place/) AND ((techno* or gerontotechnology or gerontechnolog* or digital or tablet or intelligen* or touchscreen or computer or smart or machine or numeric or virtual or monitor* or sensor* or robot*).mp or Technology/or Digital technology/ or Mobile technology or Information and communication technology/or Assistive technology/or Wireless technology/or Monitoring/or Self-Monitoring/) AND ((Aging or ageing or senior* or old* adult* or old* person* or old* people* or elder* or late life or geriatric* or gerontolog*).mp or Older adulthood/ or exp. Aging/ or Gerontology/) AND (relative* or informal carer* or caregiver* or dyad* or spouse* or famil* or support person*).mp or caregivers/ or dyads/ Limite: 2016–2021	182
Medline	(((home adj2 care) or own home or (living adj2 independent*) or (aging adj2 independent*) or (base adj2 home) or community dwelling or living alone or aging in place).mp or Independent living/or Home care services/) AND ((techno* or gerontotechnology or gerontechnolog* or digital or tablet or intelligen* or touchscreen or computer or smart or machine or numeric or virtual or monitor* or sensor* or robot*).mp or exp. technology/ or wearable electronic devices/ or hearing aids/or exp. Video Recording or Reminder Systems/or Mobile Applications/or user-computer interface/or Geographic Information Systems/or self-help devices/ or communication aids for disabled/or Robotics/or exp. Monitoring, Ambulatory/ or exp. Signal Processing, Computer-Assisted/) AND ((Aging or ageing or senior* or old* adult* or old* person* or old* people* or elder* or late life or geriatric* or gerontolog*).mp or exp. Aged/ or exp. Aging/) AND (relative* or informal carer* or caregiver* or dyad* or spouse* or famil* or support person*).mp or exp. Family/or exp. Caregivers/ Limite: 2016–2021	603
CINAHL	TIAB((home N2 care) OR “own home” OR (independent* N2 living) OR (independent* N2 aging) OR (home N2 base*) OR “community dwelling” OR “living alone” OR “aging in place”) or (MH “Home Care Equipment and Supplies”) or (MH “Home Health Care+”) AND TIAB(techno* or gerontotechnology or gerontechnolog* or digital or tablet or intelligen* or touchscreen or computer or smart or machine or numeric or virtual or monitor* or sensor* or robot*) or (MH “Technology+”) or (MH “Assistive Technology Devices+”) or (MH “Assistive Technology Services”) or (MH “Robotics”) AND TIAB (Aging or ageing or senior* or “old* adult*” or “old* person*” or old* people* or elder* or “late life” or geriatric* or gerontolog*) or (MH “Aged+”) or (MH “Aging+”) or (MH “Gerontologic Care”) or (MH “Gerontologic Nursing”) or (MH “Geriatrics”) AND TIAB(relative or “informal care*” or caregiver* or dyad* or spouse* or famil* or “support person*”) or (MH “Caregiver Support”) or (MH “Caregivers”) or (MH “Dependent families”) or (MH “Patient-Family Relations”) or (MH “Family relations”) Limite: 2016–2021	432
Web of Science	Topic((home NEAR/2 care) OR “own home” OR (independent* NEAR/2 living) OR (independent* NEAR/2 aging) OR (home NEAR/2 base*) OR “community dwelling” OR “living alone” OR “aging in place”) AND Topic(techno* or gerontotechnology or gerontechnolog* or digital or tablet or intelligen* or touchscreen or computer or smart or machine or numeric or virtual or monitor* or sensor* or robot*) AND Topic(Aging or ageing or senior* or “old* adult*” or “old* person*” or old* people* or elder* or “late life” or geriatric* or gerontolog*) AND Topic(relative* or “informal care*” or caregiver* or dyad* or spouse* or famil* or “support person*”) Limite: 2016–2021 + Document type = Article	893
AGELINE	(TI elder$ OR AB elder$ OR TI senior$ OR AB senior$ OR TI geriatric$ or gerontolog$ or ag?ing OR AB geriatric$ or gerontolog$ or ag?ing) OR (DE “Geriatric Psychiatry” OR DE “Aging” OR DE “Geriatric Education” OR DE “Gerontology” OR DE “Geropsychology” OR DE “Geriatrics” OR DE “Older Adults” OR DE “Frail Elderly” OR DE “Gerontological Nursing” OR DE “Gerontological Research” OR DE “Gerontologists” or DE “Old Old”) AND TI (caregiver$ or caregiving or family or relati$) OR (DE “Caregivers” OR DE “Long Distance Caregivers” OR DE “Long Distance Caregivers” OR DE “Care Receivers” OR DE “Caregiver Education” OR DE “Caregiving Burden” OR DE “Caregiving Rewards” OR DE “Dependent Parents” OR DE “Eldercare Programs” OR DE “Home Care Workers” OR DE “Informal Support Systems” OR DE “Respite Care” OR DE “Sandwich Generation”) OR (DE “Family Assistance” OR DE “Informal Support Systems” OR DE “Emotional Support” OR DE “Family Relationships” OR DE “Filial Responsibility”) OR (DE “Spouses” OR DE “Husbands” OR DE “Wives” OR DE “Relatives” OR DE “Adult Children” OR DE “Couples” OR DE “Daughters” OR DE “Extended Family” OR DE “Grandchildren” OR DE “Grandparents” OR DE “Great Grandparents” OR DE “In Laws” OR DE “Parents” OR DE “Siblings” OR DE “Sons” OR DE “Spouses” OR DE “Step Relatives” OR DE “Husbands” OR DE “Wives” OR DE “Couples”) AND TI (technolog$ or smart$ or monitor$ or device$ or computer$ or artificial intelligence or gerontechnology) OR (DE “Technology” OR DE “Information Technology” OR DE “Information Technology” OR DE “Automation” OR DE “Computers” OR DE “Distance Education” OR DE “Assistive Devices” OR DE “Corrective Lenses” OR DE “Durable Medical Equipment” OR DE “Hearing Aids” OR DE “Orthopedic Equipment” OR DE “Pacemakers” OR DE “Prosthetic Devices” OR DE “Monitoring Devices” OR DE “Alarm Systems” OR DE “Computers” OR DE “Artificial Intelligence” OR DE “Automation” OR DE “Computer Aided Instruction” OR DE “Computer Software” OR DE “Information Technology” OR DE “Older Computer Users” OR DE “Technology”) AND TI home or hous$ or smart house or design or living alone or aging in place OR (DE “Home Care” OR DE “Home Health Care” OR DE “Home Health Care” OR DE “Home Maintenance” OR DE “Repairs” OR DE “Home Modification”) OR (DE “Living Alone”) OR (DE “Housing Design” OR DE “Housing Improvement” OR DE “Housing Security” OR DE “Housing Characteristics” OR DE “Housing” OR DE “Housing” OR DE “Affordable Housing” OR DE “Housing Types” AND DE “Housing Characteristics” OR DE “Housing Conditions” OR DE “Housing Design” OR DE “Housing Improvement” OR DE “Housing Needs” OR DE “Housing Preferences” OR DE “Housing Security” OR DE “Residential Mobility”)	10

### Study selection

**Figure 1:**
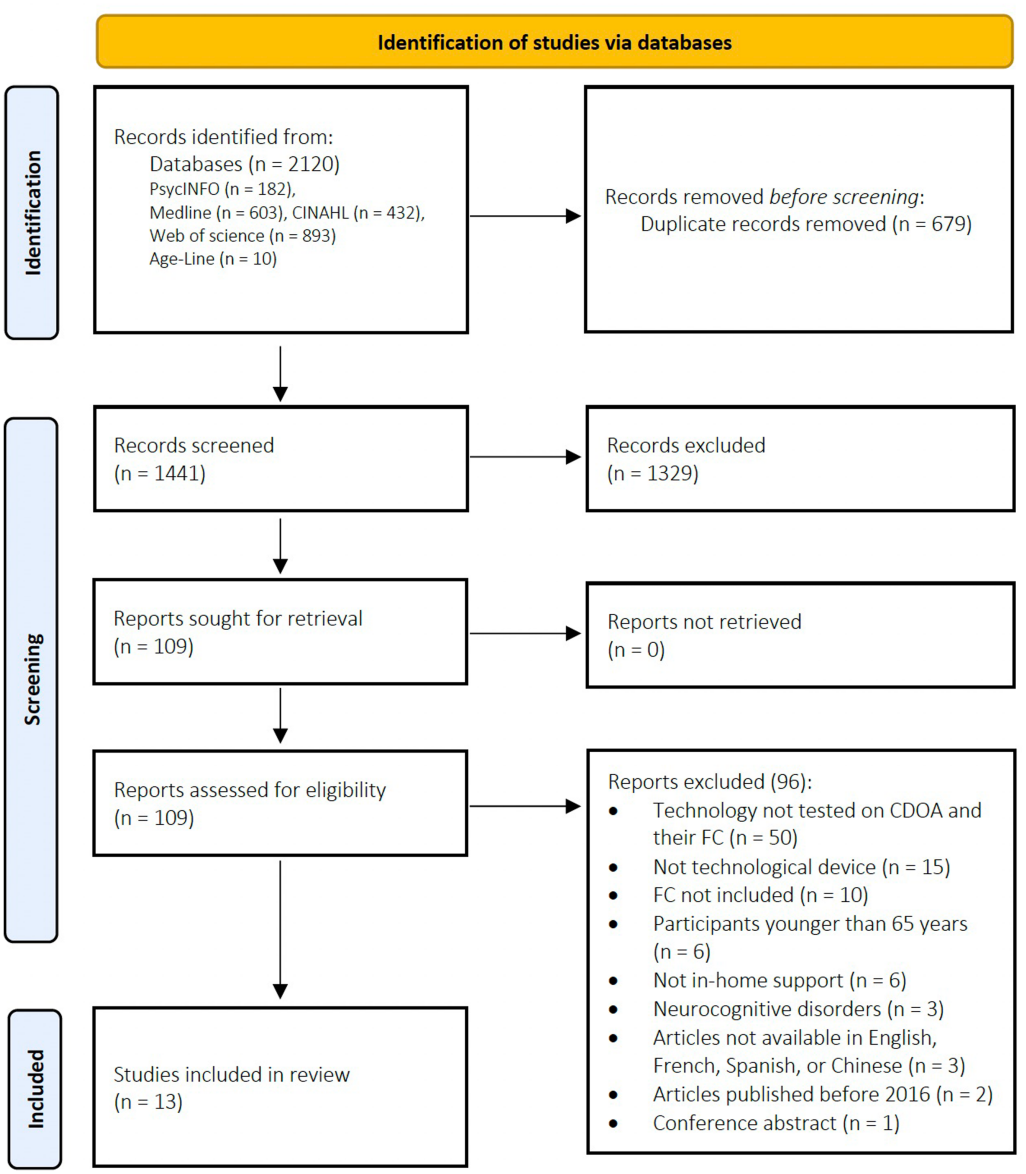
PRISMA flow diagram.

Studies were included based on the following criteria: (a) studies reporting results on the efficacy or the feasibility of gerontechnologies tested at home; (b) gerontechnologies tested with dyads of CDOA and family caregivers; (c) tested with older adults without neurocognitive impairment; (d) studies using quantitative, qualitative or mixed data analysis methods; (e) studies available in Chinese, Spanish, French, or English. Studies were excluded if: (a) the sample included older adults with a diagnosis of cognitive impairment; (b) the gerontechnology was not tested with dyads of CDOA and family caregivers; (c) the gerontechnology did not provide in-home support; (d) the article was a research protocol; (e) the studies were available in languages other than Chinese, English, Spanish, or French. As shown in Figure 1, 2,120 references were identified. Two independent reviewers separately screened titles and abstracts based on inclusion/exclusion criteria (HS and KA) using COVIDENCE software (Veritas Health Innovation, 2014). Disagreements about inclusion were resolved by the supervisor (AM). A full-text review was then conducted for the remaining 109 references (HS and KA), with a total of 13 records compatible with the inclusion criteria.

### Data extraction and synthesis

Titles and abstracts were screened by two independent reviewers (students in psychology and a student with a background in engineering) according to the inclusion/exclusion criteria. Interrater reliability was assessed as moderate for the title and abstract screening (Cohen’s kappa coefficient of 0.48). Studies matching the inclusion criteria and those being unclear regarding their eligibility were retained for a full-text review. Interrater reliability was assessed as moderate for the full-text review (Cohen’s kappa coefficient of 0.51). A third reviewer (AM) resolved eligibility discrepancies where the first two reviewers did not reach a consensus. Data extraction was conducted by two independent reviewers, and included the sociodemographic characteristics of the participants, the characteristics of the gerontechnology tested at home, the duration of the intervention with the gerontechnology, the cost, and the benefits and challenges of using each specific gerontechnology.

### Quality assessment

Three independent reviewers (HS, KA, M-CS) evaluated the quality of the empirical studies included in the present systematic review with the Mixed Methods Appraisal Tool (MMAT – Hong et al., 2018). This tool is designed for quality assessment of empirical studies included in systematic reviews. The scores range from 0 to 5, where scores near 5 indicate an excellent methodological quality. A mean score was calculated using the ratings of the three independent reviewers.

## Results

A total of 13 studies met the study criteria and were included in this systematic review. Four studies used a qualitative method (Galambos et al., 2017; Åkerlind et al., 2018; Bradford et al., 2018; Berridge et al., 2019), six studies used a mixed-methods approach (Bock et al., 2016; Suzuki and Hasegawa, 2018; Grgurić et al., 2019; Gutierrez et al., 2019; Tseng and Hsu, 2019; Corbett et al., 2021), and three studies used quantitative methods (Cohen et al 2016; Quinn et al., 2019; Pais et al., 2020). Most studies were conducted in the United States (*n* = 5) (Bock et al., 2016; Galambos et al., 2017; Berridge et al., 2019; Quinn et al., 2019; Corbett et al., 2021). Other studies took place in Switzerland (*n* = 2) (Cohen et al., 2016; Pais et al., 2020), Sweden (*n* = 1) (Åkerlind et al., 2018), Australia (*n* = 1) (Bradford et al., 2018), Chile (*n* = 1) (Gutierrez et al., 2019), Croatia (*n* = 1) (Grgurić et al., 2019), Japan (*n* = 1) (Suzuki and Hasegawa, 2018), and Taiwan (*n* = 1) (Tseng and Hsu, 2019). The collective sample of these 13 studies included 172 older adults, with a mean age of 78.5 years (SD = 7.6), and 134 caregivers with a mean age of 51.7 (SD = 7). Gender, the relationship with the family caregiver, and the caregiving situation were not systematically reported in all of the studies. For the studies reporting them, the majority included mostly females in both the older adults and family caregivers’ groups. The family caregivers were mostly daughters. Only one study reported living arrangement, specifically, that 91.2% of the participants lived alone (Cohen et al., 2016). The technologies addressed different problems including the detection of medical emergencies (Åkerlind et al., 2018), falls (Galambos et al., 2017), or health issues (Cohen et al., 2016), the early detection of difficulties performing activities at home (Bradford et al., 2018), the lack of access to information or entertainment (Corbett et al., 2021), the need for rapid action when there are behavioral anomalies in older adults’ routines (Grgurić et al., 2019), social isolation (Gutierrez et al., 2019), medication compliance (Suzuki and Hasegawa, 2018), and lack of intergenerational connection between older adults and their adult children (Tseng and Hsu, 2019).

### Level of evidence of the studies

The results of the Mixed Methods Appraisal Tool (MMAT) revealed that studies had on average a moderate quality of evidence (Table 2). The mean score obtained by the three independent reviewers was 3.7 (SD = 1). Common factors limiting the quality of the studies were linked to the sample sizes. Notably, all samples were small, a few had a high rate of attrition and a potential selection bias. For example, in Bradford et al. (2018), participants selected themselves (self-selection), thus it was mentioned that it is possible that they were already prone to positively appreciate the technology. In another study, most caregivers earned more than 100,000 US$ per year, which is considerably higher than the mean American salary (Quinn et al., 2019). Also, for the studies using a mixed methods design, divergences between qualitative and quantitative results were often not addressed (e.g., Grgurić et al., 2019).

**Table 2:** Average scores of the Mixed Methods Appraisal Tool for the studies included in the systematic review.

**Study**	**MMAT score**
Åkerlind et al. (2018)	5
Berridge et al. (2019)	5
Bock et al. (2016)	2
Bradford et al. (2018)	4.7
Cohen et al. (2016)	3.3
Corbett et al. (2021)	3
Galambos et al. (2017)	4.7
Grgurić et al. (2019)	2
Gutierrez et al. (2019)	4
Pais et al. (2020)	4
Quinn et al. (2019)	3.7
Suzuki and Hasegawa (2018)	2.7
Tseng and Hsu (2019)	4

### Description of gerontechnologies for home-support tested simultaneously with community-dwelling older adults and their family caregivers

Each of the 13 studies evaluated a different technology. The mean duration of the intervention was 11.3 months (SD = 20.3) for a total of 135.5 months of intervention combining the 13 studies. Interventions varied in terms of their duration between 1 week and 6 years. A review of these technologies and their functionalities is presented in Tables 3 and 4.

**Table 3:** Description of gerontechnologies for home-support tested simultaneously in community-dwelling older adults and their Family Caregivers.

**Authors**	**Gerontechnology name and description**	**Function**	**Price**	**Duration** **(months)**
Åkerlind et al. (2018)	eHomecare is a Swedish technology offered by the municipality that replaces home care visits including a camera for supervision at night, a videophone, and an electronic mailbox. The videophone is used for social interactions and to send reminders. The mailbox is used for reminders and to receive information.	Communication and monitoring	222$ per month	6
Berridge et al. (2019)	QuietCare includes five interconnected sensors (bathroom door, bedroom door, apartment door, refrigerator, and environmental temperature sensors). The technology detects changes in movement and informs family members and social workers based on individual norms. QuietCare is connected to a telecare call center, the family emergency contacts, and the emergency medical service when no-one can be reached.	Monitoring and communication	6 to 16$ per month	72
Bock et al. (2016)	The Lab of things (LoT) is an open-source platform that manages smart home system deployment and integration. The platform runs on a laptop in the kitchen of the older adult and connects to the cloud-side component of LoT for data storage and central management. The LoT transforms the data created by sensors (one multi-sensor and two door/window sensors at home) into sensor firing data (e.g., when a door is opened) or environmental parameters such as temperature and humidity (in the case of the multi-sensor).	Monitoring	500$	3
Bradford et al. (2018)	Smarter Safer Homes platform is a system using roughly 30 in-home sensors, different electronic medical devices (weight scales, a thermometer, and a combined blood pressure monitor and glucometer unit) connected to an iPad for self-monitoring. Sensor and medical device data are uploaded to a website (family portal) where authorized relatives can remotely monitor health and daily activities of their family member. Communication with family caregivers is also facilitated using a videoconferencing application.	Monitoring and communication	–	9
Cohen et al. (2016)	The Intelligent Wireless Sensor system allows recording the movements and activity/inactivity of the home-dwelling older adults in strategic places of their living space (e.g., living room, bedroom, bathroom, time spent in bed, and time at which the fridge was opened). The system detects changes in movements and contacts caregivers depending on the participants’ changing behavior patterns represented on a dashboard (by short message service, email, or smartphone application).	Monitoring and communication	–	-
Corbett et al. (2021)	Virtual Home Assistant is a second-generation Echo Show that had a 10.1-inch smart video screen and a third-generation Echo Dot smart speaker that was 3.9 inches in diameter and 1.7 inches high. Support persons received an Echo Spot that was 4.1 inches in diameter, 3.8 inches tall, and had a small video screen and smart speaker. The technology allows the older adult to have a voice-activated connection to the internet and receive vocal answers. It also allows videoconference communication with the primary caregiver, who received an Echo Spot.	Daily life assistance, communication	–	2
Galambos et al. (2017)	The Fall risk assessment sensor system includes a pulse-Doppler radar, a Microsoft Kinect, and two Web cameras. The system works to detect motion and falls using a machine learning approach.	Monitoring	–	24
Grgurić et al. (2019)	SmartHabits is a monitoring system using sensors to detect usual daily activity patterns. The system also contacts family members or caregivers when an unusual situation is detected. Data is stored in the Cloud Platform and used for pattern recognition and anomaly detection	Monitoring and communication	–	1
Gutierrez et al. (2019)	SocialConnector system is a PC tablet application created to facilitate family communication. The tablet is fixed on a wall or furniture inside the older adult’s house. The older adult can interact with his surroundings using voice, video, or text messaging that can be controlled using voice commands or the touch-based screen (i.e., synchronous and asynchronous voice messaging, synchronous video messaging, text messaging, and multimedia messaging). Family members receive messages from the application to invite them to engage in conversations.	Communication	–	2.25
Pais et al. (2020)	Domocare allows the monitoring of older adults using a system of ambient sensors (e.g., mobility, sleep habits, fridge visits, door events) and health-related events by wearable sensors (i.e., wearable activity tracker worn on the wrist, ECG)	Monitoring	–	12
Quinn et al. (2019)	ICMed App sends evidence-based and personalized advice based on the data collected about the health profile and the family health history of the older adults. The software is created to share information and connect older adults, family caregivers, and professional care providers.	Information and communication	–	1
Suzuki and Hasegawa (2018)	The ODP-MSS is an intelligent pill dispenser with an internal memory in which single doses of several medications intended to be taken at the same time are sealed in single film bags that are rolled onto a rotating drum. The ODP-MSS can dispense a maximum of six ODP doses per day for 60 days. The technology sends musical alerts to older adults to remind them to take medication and sends alerts to caregivers if the older adults did not take the medication. The memory stores the data that can be used by the pharmacist or physician and retrieved using a USB cable and a personal computer.	Daily life assistance and communication	–	3
Tseng and Hsu (2019)	Smart Care Interactive Systems (SCIS) is an intelligent chair that is used to monitor behaviors and heartbeat activity (e.g., user detection, heartbeat). Data are sent to a cloud and could be accessed by the caregiver using an app installed in a smartphone. The system can send alerts in case of unusual user behavior.	Monitoring and communication	–	0.25

In terms of functionalities, the technologies tested with CDOA and their family caregivers can be summarized as: (a) monitoring technologies, (b) communication technologies, (c) daily life assistance technologies, and (d) health information technologies.

**Table 4:** Characteristics and main findings of the studies included in the systematic review.

**Authors and year**	**Type of study (design)**	**Country**	**Study objectives**	**Measures**	**Results**	**User acceptance**	**Benefits**	**Difficulties using the technology**	**Adverse effets**	**Conclusion**
Åkerlind et al. (2018)	Qualitative	Sweden	To extend descriptions of how older adults with granted eHomecare and their relatives understand safety, and further to describe how they experience safety in everyday life	Interviews about the perceived sense of safety	The videophone was experienced as creating closer contact and as a tool for the older adults and their relatives to inform each other about their conditions through image and sound	Privacy concerns and fear of safety threats could affect the willingness to try a new technology	eHomecare provides economic and practical benefits concerning older adults and their relatives’ experiences of safety	Providing thorough and adequate information about the service was difficult	–	eHomecare can promote safety for older adults ageing in place and for their relatives
Berridge et al. (2019)	Qualitative	US	To examine the difference in experiences and insights of low-income, immigrant senior residents, family contacts, and staff of housing that offered a sensor-based passive monitoring system	Interviews about the how they made decisions about technology adoption or discontinuation, and about their experiences with the system	The reactions of immigrant older adults to the passive monitoring system reveal that this technology was often mismatched with their values, needs, and expectations	Asian immigrants discontinued the use at higher rate compared with other users due to fear that false alarms burden their families	Variable depending on culture	When calling to the telecare center (e.g., in case of emergency), the first response is always in English, which may cause stress to non-English speakers; the quantity of false alarms is an issue	-	Successful adoption of gerontechnologies by immigrant older populations must be culturally and practically relevant to these populations
Bock et al. (2016)	Mixed	US	To demonstrate the implementation of a smart home system using an open, extensible platform in a real-world setting and develop an application to visualize data in real time	Interview and usability questionnaire	Family members felt comfortable using the application, while older adults indicated it would be difficult to learn to use it and had trouble identifying its utility	There was a better acceptance rate from the family caregivers compared to older adults	In real time, consumers are able to view sensor events day-to-day relative to an average event level, which is useful to inform their family members, physicians, or family caregivers	Older adults had difficulties to learn to use the application and had trouble identifying utility	–	Although customization is challenging, older adults have expressed interest in smart home technologies, and one way to facilitate their adoption is through visualizations that incorporate data from smart home sensors into relevant and insightful resources
Bradford et al. (2018)	Qualitative	Australia	To seek the views and perspectives of Smarter Safer Homes (SSH) residents using in-home monitoring and explore the perspectives of relatives of SSH residents who were granted access to their relative’s activity and health data via an online portal	Perceptions on the use of technology at home and modified versions of The Microsoft’s Desirability Toolkit	Older adults experienced peace of mind from the health devices and, once accustomed, were unperturbed by the presence of sensors; there was an increase in family communication	With regards to the medical devices, residents varied in their frequency and use of them, however, all residents used at least one device once per week	To reduce the social boundaries that lead to isolation and loneliness in older adults, providing peace of mind to family caregivers	The iPad was found to be the most challenging component of the platform and inaccuracies in sensor data and difficulties in sensor placement proved frustrating for residents and researchers	Minor annoyances derived from sensor placement and function	There was an overall positive response to the system, despite a slight tendency for residents to modify their behavior due to perceived surveillance
Cohen et al. (2016)	Quantitative	Switzerland	To explore the acceptability (usefulness, satisfaction, ease of use, and intention to use) of an intelligent wireless sensor system (IWSS) among home-dwelling older adults	The Resident Assessment Instrument for Home Care, Confusion Assessment Method, Cognitive Performance Scale, Geriatric Depression Scale, Informed Questionnaire on Cognitive Decline in the Elderly, and acceptability of the IWSS using a home-made questionnaire	Both older adults and their family caregivers considered the performance and usefulness of the IWSS intervention to be low to moderate and the majority of the participants were unsatisfied with its ease of use, while their informal caregivers were more satisfied with the program	Only 26.1% of older adults and 53.3% of their family caregivers were satisfied, but participants felt that the IWSS was intrusive and that they were being watched	One-third of older adults and three-quarters of family caregivers considered the IWSS useful for older adults who wished to remain in their homes, and believed that the IWSS was an appropriate means of ensuring safety in case of falls	Family caregivers were dissatisfied with the need to acknowledge each alarm message with a telephone call, some rural areas were not always covered by the mobile phone network, and one-fifth of the participants or informal caregivers contemplated leaving the study	The perception of intrusiveness went so far as to create conflicts between participants and their family caregivers	IWSS programs installed were not always easy to use and generally demonstrated only low-to-moderate acceptability and the IWSS failed to precisely and rapidly detect every health issue in daily life
Corbett et al. (2021)	Mixed	US	To describe virtual home assistant (VHA) use and usefulness from the perspective of older adults and their support persons	PROMIS Global Scale for older adults, Caregiver Burden Scale for family caregivers and qualitative telephone interviews	Older adults and their support persons used the VHAs in similar ways to request information, listen to music, obtain weather forecasts, and enjoy other types of entertainment (e.g., jokes and podcasts)	Participants did not have privacy concerns about using a VHA	Benefits included the hands-free feature of the VHA and security by allowing older adults to contact someone in an emergency and the facilitation of interactions throughout the day reducing isolation (e.g., video calling feature)	Challenges integrating Alexa with other tools that served similar purposes and older adults’ needs for more education and training about the capabilities of VHAs (e.g., “Getting Used to Another Device”)	–	Participants used the VHA regularly over time, primarily for information, entertainment, or to receive prompts; while future desired uses included the health promotion and management of their health conditions
Galambos et al. (2017)	Qualitative	US	To explore the perceptions and preferences of older adults and their family members about a fall risk assessment system (FRAS)	Individual face-to-face interviews with older adults and face-to-face or telephone interviews with family caregivers of participants were made during the installation phase and at 6, 12, 18, and 24 months	Positive perception at the preinstallation phase (e.g., feelings of security), at 6 months (e.g., strong connection to their own health information), 1 year (e.g., appreciation of feedback and reports), 18 months (e.g., no interference with daily routine), and 24 months (e.g., sense of serving purpose)	Over time, the FRAS became a normal part of the environment, no longer a novelty, and was eventually accepted as a helpful device within one’s living environment	The FRAS was regarded as a tool that helped increase their safety and activity level and served as a motivator to do better	The aesthetics of the system was mentioned as something that could be improved; some users did not like the color, location, or style of the wooden box that held the equipment	-	Sensor monitoring was regarded positively by both older adults and family caregivers, and as a means to hold on to independence to age in place
Grgurić et al. (2019)	Mixed	Croatia	To evaluate if the prototype system could successfully learn typical daily patterns, detect unusual situations in the household of the older person living alone, and notify family caregivers when an unusual situation is detected	Data analysis of the number of detection and amount of patterns learned. Usability questionnaire and a free form question (i.e., *“Is there anything else you would like to share about the system?”*)	During the pilot and testing phase, using six sensors the system was able to learn on average 23 patterns per single household in the first 30 days of the usage.	Older adults perceived that this technology is usable as they did not have to interact with the system explicitly or compromise their privacy, while family caregivers interacted with the system explicitly and received notifications if something unusual happened in the older adult’s household	The home users, in general, liked the system based on the use of simple off-the-shelf sensors that do not invade privacy without explicit interaction of home users	There was no problem with the autonomy of the sensors, but for more prolonged and more extensive use, this would be an issue; the automatic resets of the Internet connection in the mobile Wi-Fi routers posed some disturbance without affecting the core-system functionality	The light coming from the hardware was sometimes too distracting	The proposed system can easily improve the quality of care with simple smart-home sensors that can provide essential and continuous information about the status of the occupant and the environment
Gutierrez et al. (2019)	Mixed	Chile	To evaluate the effect of introducing the SocialConnector system at the home of a sample of older adults, in the interaction with their family network	Data from automatically generated system usage logfiles pre-intervention (weeks 1–3), during the intervention (weeks 4–6), and post-intervention (weeks 7–9)	Mediating the interaction of family members with notification triggers does have an effect on the volume of calls, messages, and photos sent to the older adults	Older adults using SocialConnector did show increased social engagement, particularly with family members, when exposed to interacting with the system over a period of 9 weeks	The system involves the entire family network in the process	Major concern involving privacy matters and information disclosure across the family network and reticence on trusting the technology for mediating intergenerational communication about personal matters	–	This study proposes 20 recommendations that positively impact the usability of the devices, which consider not only the requirements of elderly people as part of the aging in place process, but also the typical capabilities and restrictions of the rest of the family members that support the process
Pais et al. (2020)	Quantitative	Switzerland	To evaluate the usability, functionality, and effects of a new in-home monitoring system—combining ambient and wearable sensors—among home-dwelling older adults, their family caregivers, and nurses for the support of home care	Semistructured interviews face-to-face or phone calls based on the French version of the Quebec User Evaluation of Satisfaction with assistive Technology (QUEST) for older adults and the caregiver quality of life scale for family caregivers	The majority of participants considered that in-home sensors were helpful (ambient and wearable) with more favorable opinions toward ambient sensors than toward Activity tracker, and ECG	The majority of older adults and family caregivers reported that they would like to continue using in-home sensors in case of insurance reimbursement	To help staying at home improving home care, preventing domestic accidents, and reducing family stress	Improvements of the technology could include the design of sensors that are smaller, lighter, and more user-friendly and comfortable for older adults, as well as advances in machine learning for detection of specific events at home	–	Overall, the opinions of older adults, family caregivers, and nurses were positively related to in-home sensors, but nurses were less enthusiastic about their use in clinical practice
Quinn et al. (2019)	Quantitative	US	To determine the usability of a mobile App in a community-based older adult population aged ≥65 years	Participant engagement was measured by weekly surveys sent via an App push notification, the quality of the App, and Usability	In fourth week post-intervention, 60% of participants were aware of their health conditions, 40% wanted to learn or felt motivated to take care of their health, and half of family caregivers indicated they wanted to use the App to manage health appointments, records, and share health information	While technology use was common in the cohort among well-educated older adults, engagement with the mobile App was average	The App may be used for older adults to improve participation in health care decisions made by family caregivers and providers, to self-manage health and social needs, and to improve engagement and social connections	Technical issues, including, but not limited to, log in and connectivity issues, discouraged participants and delayed or limited use, likely leading to loss to follow-up	–	Technology use is high among this population despite low participant usability and engagement
Suzuki and Hasegawa (2018)	Mixed	Japan	To evaluate a one-dose package medication support system (ODP-MSS) for medication support and telecare home monitoring of older adults	Interviews with older adults and family caregivers about missed medication and data log of medications taken or not taken, as well as automatic calling, from the memory of the ODP-MSS	Most older adults had 100% medication adherence and those who had missed doses due to forgetfulness took medicine after the caregiver called	Participants reported that the ODP-MSS provided a useful reminder to take medicine at the time of the alert	The device provided a useful reminder to take medicine, the caregiver’s call was useful as a telecare home monitoring system, and older adults who had missed doses due to forgetfulness took medicine after the medication supporter called	Limitations included device jamming, patients feeling obligated to stay home during medication administration times as they could not take the medication when they left their homes, supporters receiving too many calls, and an irregular lifestyle interrupted routine taking of medications (e.g., ODP-MSS did not match their actual mealtimes)	-	The technology helped prevent missed doses resulting from older adults’ forgetfulness and may serve as a useful component of telecare home monitoring for elderly people living independently at home, to reduce the burden associated with medication support, and to prevent medication errors
Tseng and Hsu (2019)	Mixed	Taiwan	To explore the use of a smart care interactive system with a chair (SCIC) to improve the intergenerational relationships at home	Intergenerational Relations Scale (IRS), usability questionnaire, and interviews	The SCIC was shown to significantly improve the emotional support and parent–child interactions with the elderly as well as the intergenerational relationships (e.g., parent–child interactions)	Well accepted, despite design limitations	Through the App, visual information display, and warning messages family caregivers are able to effectively understand the elderly’s active and rest status	The design of the back of furniture is not long enough, the elderly users are dissatisfied with the neck support part, and it is very difficult to move the footrest that sometimes hinders the movement of elderly people in the living room	–	The interactive chair can significantly help the elderly in terms of emotional support and parent–child interactions (e.g., care about older adults’ health and facilitation of interactions between older adults and family caregivers)

#### Monitoring technologies

Monitoring technologies are developed for supervision and to allow rapid detection of anomalies or dangers at home to ensure the safety of older adults. Most technologies (10/13) included a system to monitor individuals or the environment (Bock et al., 2016; Cohen et al., 2016; Galambos et al., 2017; Åkerlind et al., 2018; Bradford et al., 2018; Suzuki and Hasegawa, 2018; Berridge et al., 2019; Grgurić et al., 2019; Tseng and Hsu, 2019; Pais et al., 2020). Cameras and sensors were the most widely used monitoring technologies in these studies. Cameras were used to monitor older adults in bed during nighttime (Åkerlind et al., 2018) and to detect falls while older adults were walking in their homes (Galambos et al., 2017). Sensors were used to detect motion and record usual activity patterns (Bock et al., 2016; Cohen et al., 2016; Bradford et al., 2018; Berridge et al., 2019; Grgurić et al., 2019; Pais et al., 2020). For example, the Intelligent Wireless Sensor System (IWSS) consists of a set of sensors that record older adults’ movements in different rooms of their homes. Messages are sent to family caregivers when there is a behavioral pattern modification (Cohen et al., 2016). Four technologies used a system of alerts to signal anomalies (e.g., fall detection or change in walking pattern) (Galambos et al., 2017; Berridge et al., 2019; Grgurić et al., 2019; Tseng and Hsu, 2019) or to confirm that an activity has been performed by the older adult (e.g., self-administration of medication) (Suzuki and Hasegawa, 2018). Alerts were sent by the system and received by the family caregivers through text messaging and phone calls.

#### Communication technologies

A few technologies are designed to enhance the communication between the older adult living at home and their family caregivers. Five studies presented technologies serving this goal (Åkerlind et al., 2018; Bradford et al., 2018; Gutierrez et al., 2019; Quinn et al., 2019; Corbett et al., 2021). Smarter Safer Homes (Bradford et al., 2018) and the ICMed technology (Quinn et al., 2019) allowed the sharing of information about the health and daily activities between the older adult and the family caregiver via a platform. Finally, four technologies included a system of communication via phone calls, videoconferencing and/or text messages to connect older adults with their social circle (Åkerlind et al., 2018; Bradford et al., 2018; Gutierrez et al., 2019; Corbett et al., 2021). This allowed family caregivers to communicate in real time, do check-ups, and provide reminders when needed.

#### Daily life assistive technologies

A few technologies are designed to assist older adults in their daily life. These technologies include any electronic tool or equipment designed to help a person perform their regular daily activities, such as cooking, cleaning, entertaining, or planning. Two technologies served this purpose, namely the Virtual Home Assistant and the One-dose package medication support system. The Virtual Home Assistant is an electronic tablet used for entertainment, information search, planning (e.g., access to the calendar), and communication (Corbett et al., 2021). The One-dose package medication support system was used to help with medication compliance (Suzuki and Hasegawa, 2018).

#### Health information

Some technologies or applications are developed to increase access to evidence-based information that can help both older adults and their family caregivers manage their health and well-being. *ICMed* is a mobile application serving this goal. It uses the information collected on the older adult and their families to generate personal health advice (Quinn et al., 2019).

### Benefits using gerontechnologies

Gerontechnologies have the potential to help CDOA maintain their autonomy and age in place when they are developed to respond to the specific needs of dyads. A review of the benefits found in the studies included: (a) increased communication and family participation, (b) increased sense of safety, (c) reduced stress of family members and CDOA, and (d) other perceived benefits.

#### Increased communication and family participation

Several studies revealed that the use of technology improved the communication between older adults, their family caregivers, and health care professionals (Åkerlind et al., 2018; Bradford et al., 2018; Quinn et al., 2019; Tseng and Hsu, 2019; Corbett et al., 2021). For instance, during an interview, a family caregiver who used the system Smarter Safer Homes shared the benefits of videoconferencing to assess the mood and state of the older adult (Bradford et al., 2018). Gutierrez et al. (2019) also showed that the Social Connector system can facilitate the interaction of family members and that video-calls were a highly appreciated activity by older adults. Tseng and Hsu (2019) found that the use of the Smart Care Interactive Systems (SCIS) with a chair significantly improved the quality of parent–child interactions. Hence, these technologies have the potential to decrease loneliness, by connecting older adults to their social network and improve the quality of relationships.

#### Increased sense of safety

Four studies revealed that in-home monitoring had a positive influence on feelings of safety (Cohen et al., 2016; Galambos et al., 2017; Åkerlind et al., 2018; Pais et al., 2020). For instance, older adults viewed the eHomecare system as a valuable resource to ensure safety. Family caregivers felt relief knowing that the technology was in place, because it provided information that the older adult was out of danger. It made it easier to keep balance with other responsibilities and social life (Åkerlind et al., 2018). Galambos et al. (2017) found that both family caregivers and older adults perceived an increased sense of safety using the Fall risk assessment sensor system. The Intelligent Wireless Sensor system was perceived as useful to ensure safety at home in case of falls by 34.8% of older adults and by 76.5% of family caregivers (Cohen et al., 2016). A higher proportion of older adults (74.5%) and a similar rate of family caregivers (70%) viewed the technology Domocare as useful to prevent falls and increase quality of life (Pais et al., 2020).

#### Reduced stress of family members and community-dwelling older adults

Improvements in communication and monitoring of potential threat is associated with stress reduction in older adults and the perception that gerontechnologies are useful to reduce family stress (Åkerlind et al., 2018; Bradford et al., 2018; Pais et al., 2020). For instance, family caregivers who used the eHomecare system noticed a decrease of concerns regarding the safety of the older adult. A total of 83% of older adults who used the Smarter Safer Homes system experienced peace of mind during the intervention (Bradford et al., 2018). Finally, older adults and family caregivers perceived that the use of Domocare could help reduce family stress by increasing the supervision of the older adult (Pais et al., 2020).

#### Other perceived benefits

In two studies, CDOA felt motivated to take better care of their health, after using technologies, such as the Fall Risk Assessment System (Galambos et al., 2017) and the ICMed Application (Quinn et al., 2019). Furthermore, the study on ICMed application showed that communication technologies have the potential to increase the participation of the older adult and their family caregivers in decisions regarding health (Quinn et al., 2019). Finally, the One-dose package medication support system was reported to be useful to compensate for forgetfulness and increase medication adherence (Suzuki and Hasegawa, 2018). All these benefits promote aging in place. However, several challenges need to be addressed to optimize the implementation of these technologies.

### Challenges using gerontechnologies

Challenges and negative opinions have also been expressed by CDOA and their family caregivers. Their feedback is crucial for the development of gerontechnologies to be used at home that suit the profile, the preferences, and the needs of the dyads. Challenges included: (a) difficulties using the technologies, (b) technical problems, (c) privacy issues, (d) increased stress and dissatisfaction, and (d) a mismatch between values and needs.

#### Difficulties using the technologies

The use of technological devices often requires learning new skills. CDOA reported that learning how to correctly use technologies is a challenge (Bock et al., 2016; Bradford et al., 2018; Corbett et al., 2021). For example, older adults reported having difficulties learning how to use the technology and to identify its purpose (Bock et al., 2016). Participants reported that explaining how the data collected can be helpful to family caregivers and physicians would help them better understand their utility. It was also suggested that simplifying the visualization provided by the sensor system and demonstrations with case examples could increase its usability. In another study, older adults reported that it was challenging to adapt to a new device, especially when it served the same purpose as another technology already available in their homes (Corbett et al., 2021). Therefore, training was identified as an important need for technological implementation in older adults. Another study showed that the use of an iPad was perceived as difficult for a few older adults because of the visual and motor skills required, as well as lack of familiarity with the technology and its capabilities (Bradford et al., 2018). This is compatible with other studies with smartphones and tablet use in older adults (Barnard et al., 2013; Wilson et al., 2022).

#### Technical problems

Three studies reported technical problems during the intervention phase (Cohen et al., 2016; Grgurić et al., 2019; Quinn et al., 2019). First, some connectivity problems with mobile phone network occurred in rural areas while using Intelligent Wireless Sensor System (IWSS) (Cohen et al., 2016). Hence, some family caregivers did not receive the alarm messages. Connectivity issues were also found using the ICMed Application, along with log in problems (Quinn et al., 2019). Variability in the Internet connectivity caused family caregivers to receive system-offline notifications (Grgurić et al., 2019). The prolonged use of technologies like the SmartHabits system requires a change of batteries for the sensors. Battery replacement needs planning to make sure that the technology will be constantly operating at home.

#### Privacy issues

The perception of intrusiveness and the discomfort regarding loss of privacy is part of the downsides of environmental or personal monitoring reported by older adults (Cohen et al., 2016; Åkerlind et al., 2018; Gutierrez et al., 2019). Privacy concerns have been reported by participants in two studies (Åkerlind et al., 2018; Gutierrez et al., 2019). Feelings of being watched were experienced by older adults using the Intelligent Wireless Sensor System and triggered conflicts in some families (Cohen et al., 2016).

#### Increased stress and dissatisfaction

The use of gerontechnologies has a different impact on the level of stress and satisfaction. For instance, frequent false alarms can increase stress in family caregivers (Berridge et al., 2019). A few family caregivers felt annoyed by the number of alarms and calls needing to be answered (Cohen et al., 2016; Suzuki and Hasegawa, 2018). For older adults in an emergency situation, language barriers in communication while interacting with an employee of the Telecare Center can be a stressful experience for non-English speakers (Cohen et al., 2016).

#### Mismatch between values and needs

Studies showed that the use of technology can sometimes create a mismatch between values and needs (Suzuki and Hasegawa, 2018; Berridge et al., 2019). For instance, technologies might not match the expectation of how the older adult wished to be cared for by family members and sometimes led to conflict with family caregivers. Devices like the ODP-MSS did not allow enough flexibility in the medication administration (e.g., the older adult could not take their medication if they were away from home). As a consequence, a few older adults felt obligated to remain at home. Furthermore, four out of nine older adults perceived the technology as not useful because they could take medicine without relying on the ODP MSSS (Suzuki and Hasegawa, 2018).

## Discussion

The goal of this systematic review was to summarize the research findings on in-home interventions using gerontechnologies tested simultaneously with CDOA with unimpaired cognition and their family caregivers. More specifically, we aimed to describe the technologies, their benefits and challenges, and the evidence level of the studies about them published between 2016 and 2021. We also aimed to provide recommendations for technological development, implementation, and research. To our knowledge, this is the first study synthesizing the evidence concerning the efficacy of technologies designed to support CDOA-family caregiver dyads. The review was conducted to inform older adults, family caregivers, healthcare professionals, scientists, and policymakers about the gerontechnologies available to enable them to make well-informed decisions on their use and development.

Surprisingly, we found only 13 studies meeting our eligibility criteria. The technologies were usually tested on a small sample of participants and were designed to monitor older adults, promote communication between older adults and family caregivers, help with daily tasks, and provide useful information that can be used to optimally manage their health. Most studies were conducted in the United States. Only four studies were conducted in Europe and two in Asia, even though these continents represent most of the world’s oldest population (United Nations, 2021). The majority of studies did not specify the price of the technology used, preventing people from making decisions based on the cost/efficacy. Large differences were found related to the intervention duration, ranging from 1 week (Tseng and Hsu, 2019) to 6 years (Berridge et al., 2019). The quality of studies also varied greatly, with mixed and quantitative studies receiving lower scores due to their small sample size and risk of non-response bias. Given their position in the development process, it is common for these studies to have small sample sizes. They are often the first step before conducting large implementation studies. In general, pilot studies allow for iterations to refine the technologies being tested. To our knowledge, none of these 13 studies has moved to a wider implementation phase.

Several benefits have been reported by dyads of CDOA and family caregivers, such as an increase in communication and feelings of safety. However, some gerontechnologies elicited different reactions in older adults and family caregivers, including reports of technical difficulties, learning challenges, emotional reactions (e.g., increased stress), and interpersonal difficulties (e.g., family conflicts). These differences in reaction can perhaps be addressed via co-designing technologies to facilitate their development, increase confidence levels in their use and efficacy.

The results of a systematic review of assistive technologies in dementia care showed similar results with good acceptance to facilitate daily living (Pappadà et al., 2021). Although they included intervention studies (e.g., telemedicine) and a different population (i.e., people with dementia), the potential of technology is clearly to provide monitoring and security of older adults, support in activities of daily living, and psychosocial support. The use of these technologies seems to be increasing and they can be very useful during future pandemics. Taken together, gerontechnologies provide concrete support to older adults and family caregivers when they respond to specific needs and the different problems that can be experienced in the continuum ranging from normal aging to dementia.

Currently, education about gerontechnologies and their efficacy is needed to inform the general population, clinicians, and policy-makers about the options available to promote independent living in the older adult population. Innovative solutions to quickly test, implement, and commercialize these technologies remains a challenge as there is a gap between their development and community implementation. Initiatives to educate the public in the availability of these technologies and promote research are currently underway (Aboujaoudé et al., 2023; envisAGE, https://www.envis-age.ca/en/). Still, the lack of evidence on their efficacy impedes informed decision-making. We provide the following recommendations based on the current systematic review to address some specific challenges preventing the use and dissemination of these gerontechnologies.

### Recommendations for technological development, implementation, research, and public policies

#### Technology development

1. To develop gerontechnologies that are sensitive to the need for privacy of older adults. Privacy is an important ethical issue that must be considered during the development of gerontechnologies (Sundgren et al., 2020). For example, studies reported that cameras are less accepted because they are perceived as more intrusive (Boström et al., 2013; Claes et al., 2015). Alternative methods seem to be more appreciated by older adults (e.g., sensors for movement detection or wearable technologies for fall detection instead of cameras or microphones).
2. To develop technologies that are easy-to-use. Technologies that are easy-to-use can increase their acceptability. Also, considering potential physical, sensorial, and physical barriers in the development of gerontechnologies is a crucial step to make them more inclusive.
3. To develop technologies that respond to unmet needs at home. It is important that the functionalities respond to unmet needs identified through a co-construction process as it influences the perceived usefulness of gerontechnologies, which has been linked to positive attitudes towards their use (Chen and Chan, 2014). It is understandable that older adults would prefer using older technologies already in place instead of replacing them with new ones, since it does not require any adaptation or financial outlay.

#### Implementation

1. To provide training and guided practice to CDOA to help them learn new skills. Training was identified as a need in a few studies (Bock et al., 2016; Bradford et al., 2018; Corbett et al., 2021) as lack of it is a barrier to technology adoption. Training facilitates learning new skills and helps overcoming barriers to utilization of new technologies and devices (Chen and Chan, 2014). Strategies recommended include training by healthcare professionals, providing video or written instructions as well as providing access to continuous technical support. Also, providing a test period without penalty could allow older adults to explore the technologies before purchasing them to make sure that they really respond to their needs.
2. To evaluate the needs, the values, and the preferences of family caregivers and older adults simultaneously and explore the options available to the dyads. It is important that clinicians provide information about the interventions currently available and listen to the preferences of families. It is important to explore different alternatives to solve problems, such as forgetfulness, isolation, or mobility issues. Needs exploration can help families make informed choices and increase their feeling of self-determination, which is important to promote psychosocial health (Ntoumanis et al., 2021). Also, personalizing the interventions can ensure optimal results in CDOA and their family caregivers in their unique social, economic, and environmental context (Ebrahimi et al., 2021).
3. To ensure that the intervention proposed matches the current physical and cognitive autonomy level of the older adult. It has been found that interventions are perceived as more acceptable depending on the perception of the benefits of the technology. For instance, older adults are more likely to accept monitoring technology when experiencing mobility issues if it allows them to stay in their homes. In contrast, feelings of being able to perform a daily activity without the technology can lead older adults to perceive it as not useful (Tseng and Hsu, 2019). More importantly, it can have negative consequences on older adults, such as increasing their feeling of becoming dependent on the technology to do something that they can still do without it.

#### Research

1. To adopt a co-construction approach. To maximize the agency of older adults and their family caregivers and to ensure that the interventions match their values and needs, we encourage the active consultation and participation of community stakeholders in research on development and adaptation of gerontechnologies (Closon and Léonard, 2016). Researchers are encouraged to describe the phases of development of their technologies, including the co-construction process and the persons involved in the different iterations.
2. To document the effects of interventions on quality of life, well-being and other psychological outcomes in CDOA and their family caregivers. In the majority of the studies included in this systematic review, these effects were not documented and would provide additional evidence of the benefits of gerontechnologies for the dyads of CDOA and their family caregivers. Personal variables are important in technology adoption.
3. To conduct scaled evaluation and implementation. Future studies must evaluate the effectiveness of interventions using gerontechnology with end-users and clinicians, in real-world contexts (e.g., integration in current psychosocial or nursing interventions). These studies should include different contexts to generate evidence of generalizability (e.g., different populations of older adults and geographical locations). Conducting randomized control trials with bigger samples of CDOA and family caregivers is not always possible considering the costs both of the technological development itself and of the research. However, alternative research methods can be used. For instance, interrupted time series or single pretest-post designs can be used (Wang et al., 2021)

#### Public policies

1. To give access to information and training on gerontechnologies to managers and healthcare professionals. This step is important to facilitate implementation of gerontechnologies for home support in different organizations (e.g., the healthcare system). University curricula need to include more training in technology, rehabilitation, and older adults’ needs.
2. To fund studies evaluating the cost-effectiveness of interventions using gerontechnologies. This recommendation is based on the absence of studies evaluating the cost-effectiveness of interventions using gerontechnologies for CDOA and family caregivers. This type of study is crucial to influence future governmental investments for home support.

## General conclusion

This systematic review identified gerontechnologies that have been tested to support aging in place among among CDOA without cognitive impairment and their family caregivers. It provided information on the benefits and challenges perceived by the dyads, the quality level of the studies included, and some recommendations to address challenges linked to dissemination and implementation of these technologies.

Gerontechnologies are an innovative solution to help older adults age in place and maintain their autonomy and independence. Efforts must be made by scientists, healthcare professionals, and policy-makers to make these interventions accessible and adapted to the specific challenges encountered by older adults and their families.

## Data availability statement

The original contributions presented in the study are included in the article/supplementary material, further inquiries can be directed to the corresponding author.

## Author contributions

All authors listed have made a substantial, direct, and intellectual contribution to the work and approved it for publication.
